# Assessment of Ulcerative Colitis Patients with Elevated Neutrophilic Infiltration in the Colonic Mucosal Epithelium Using the Komagane Subclassification of the Geboes Score Grade 3

**DOI:** 10.3390/jcm14155180

**Published:** 2025-07-22

**Authors:** Satoshi Ukai, Ichitaro Horiuchi, Tsuyoshi Terashima, Kaori Horiuchi, Akira Horiuchi

**Affiliations:** 1 Digestive Disease Center, Showa Inan General Hospital, Komagane 399-4117, Japan; ukai.satoshi.3104@gmail.com (S.U.); koraki648@yahoo.co.jp (K.H.); 2 Department of Gastroenterology, Shinshu University School of Medicine, Matsumoto 390-8621, Japan; ichitaro617@yahoo.co.jp; 3 Department of Pathology, Showa Inan General Hospital, Komagane 399-4117, Japan; tera.tsuyo@gmail.com

**Keywords:** ulcerative colitis, IL-23, neutrophilic infiltration, Geboes score

## Abstract

**Background:** Interleukin (IL)-23 exerts its effects by activating Th17 cells, resulting in high neutrophilic infiltration in the colonic mucosal epithelium. We have developed a scoring method for refining the Geboes score Grade 3 to identify active ulcerative colitis (UC) patients with high epithelial neutrophilic infiltration (Geboes Grade 3.2 or 3.3). **Methods:** Colonoscopy and histology findings were assessed using the Mayo endoscopic subscore (MES) and the Geboes score Grade 3. The percentage of crypts with neutrophilic infiltration, which was calculated as the number of crypts with neutrophilic infiltration/total crypts in a glass slide, was used to subclassify the Geboes score Grade 3 into Grades 3.0, 3.1, 3.2, and 3.3. **Results:** This scoring method was then applied to 30 enrolled patients (20 men; median age: 46 years), yielding the following distribution: Geboes Grade 3.0 in six (20%) patients, Grade 3.1 in seven (23%) patients, Grade 3.2 in sixteen (53%) patients, and Grade 3.3 in one (3%) patient. Of the 18 UC patients with MES 2, 5 (28%) were classified as Grade 3.1 and 12 (67%) were classified as Grade 3.2. One of the IL-23 antagonists, mirikizumab treatment, resulted in clinical and endoscopic improvements in 10 active UC patients who were classified as Geboes score ≥ 3.2. **Conclusions:** We developed a novel Geboes score Grade 3 scoring method and applied it to 30 patients; approximately 60% were classified as Grade 3.2 or higher. This method may help to identify UC patients who are likely to respond effectively to IL-23 antagonists.

## 1. Introduction

The treatment strategy for ulcerative colitis (UC) has evolved based on the treat-to-target approach, which emphasizes achieving predefined therapeutic goals to improve long-term patient outcomes [1]. In recent years, the number of therapeutic agents for UC with distinct mechanisms of action has expanded rapidly, reflecting advances in the understanding of UC pathophysiology and immune modulation. Consequently, there are now various treatment options available for active UC. This expansion in therapeutic choices has led to a more complex decision-making process for both patients and clinicians when selecting the most appropriate treatment strategy [2,3]. Among the immunological pathways involved in UC, interleukin-23 (IL-23) has been recognized as playing a key role in disease pathogenesis, particularly through its interactions with neutrophils [4]. IL-23 exerts its effects by activating Th17 cells, which, in turn, activate neutrophils. Neutrophilic infiltration into the colonic mucosal epithelium plays a critical role in tissue damage and inflammation in UC, making it a crucial histopathological marker for disease activity assessment [4,5].

The Geboes score is a widely used histological grading system for UC, and a Grade 3 score specifically focuses on the presence and extent of neutrophilic infiltration in the colonic mucosal epithelium [6]. We have been exploring methods to identify patients with active UC who present with IL-23 activation. However, one of the major challenges associated with the Geboes score is interobserver variability among pathologists, which leads to inconsistencies in histological assessment, particularly in grades other than Grade 3 and 4 [7]. Furthermore, the original description of the Geboes score did not indicate how to specifically evaluate the Geboes score Grade 3 [6]. This study aimed to develop a simplified and clearly defined scoring method based on the definition of the Geboes score Grade 3, with the goal of enhancing the consistency and reproducibility in histological assessment among pathologists. Additionally, we attempted to prospectively identify active UC patients with Grades 3.0, 3.1, 3.2, or 3.3 using this scoring method.

## 2. Materials and Methods

### 2.1. Patients and Study Design

This was a prospective study conducted at Showa Inan General Hospital (Komagane, Japan) between February 2024 and January 2025. A total of 30 patients with active UC who underwent colonoscopy with histopathological examination were included. Among them, some patients had recurrent UC, while others were newly diagnosed with UC. Patients were diagnosed with UC based on the standard diagnostic criteria combining endoscopic and histopathological findings [6,8,9]. Patients with indeterminate colitis or Crohn’s disease were excluded. Blood tests and colonoscopy with histology were performed before enrollment in this study; we assessed the clinical activity (clinical activity index (CAI) and numeric rating scale for bowel urgency), endoscopic activity, and pathological activity. The CAI used in this study was the Rachmilewitz Clinical Activity Index [8].

The Ethics Committee of Showa Inan General Hospital reviewed and approved the study protocol (No. 2023-7). All patients provided their written informed consent for participation prior to their enrollment in the study. This study adhered to the tenets of the Declaration of Helsinki.

### 2.2. Endoscopic Evaluation

The colonoscopy findings were assessed using the Mayo endoscopic subscore (MES) [9] as follows: MES0—normal or inactive disease; MES1—mild disease (erythema, decreased vascular pattern, and mild friability); MES2—moderate disease (marked erythema, absent vascular pattern, friability, and erosions); and MES3—severe disease (spontaneous bleeding and ulceration). Biopsies were obtained from the most inflamed area, which was identified during colonoscopy, corresponding to the site used for MES evaluation.

### 2.3. Histopathological Evaluation

A total of 95 hematoxylin and eosin (H and E)-stained slides were analyzed. Multiple slides per patient were evaluated to ensure the comprehensive sampling of the colonic mucosa. Two clinicians independently assessed the histological samples—one was an experienced pathologist and the other was a gastroenterologist with experience in histopathological evaluation. The Geboes score Grade 3 was used to evaluate the presence and extent of neutrophilic infiltration in the colonic mucosal epithelium in active UC patients [6].

### 2.4. Komagane Evaluation Method of the Geboes Score Grade 3

**Figure 1:**
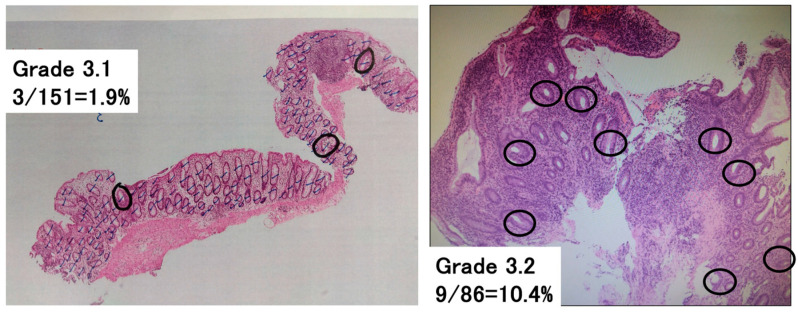
Assessment of Geboes score Grade 3. The percentage of crypts with neutrophilic infiltration was calculated as the number of crypts with neutrophilic infiltration/the total number of crypts on a glass slide. This was used to subclassify the Geboes score Grade 3 into Grades 3.0, 3.1, 3.2, and 3.3. Neutrophilic infiltration was defined as the presence of at least two neutrophils in the crypt lumen. The black lines indicate the counted crypts, while the circles denote crypts with neutrophilic infiltration. Left—Grade 3.1 (3/151 = 1.9%). Right—Grade 3.2 (9/86 = 10.4%).

To assess the Geboes score Grade 3, which evaluates neutrophilic infiltration into the epithelium, the following methodology was newly developed (Figure 1). The total number of crypts on one glass slide was counted, and then the number of crypts with neutrophilic infiltration was counted. Crypts with neutrophilic infiltration were defined as those comprising at least two neutrophils, which contain cytoplasmic neutral pink granules, within the crypt lumen. The number of crypts with neutrophilic infiltration/the total number of crypts per glass slide was calculated and used to classify patients with a Grade 3 score into groups with a Geboes score of Grade 3.0, 3.1, 3.2, or 3.3. Both cross-sectional and longitudinally sectioned crypts were counted, while the superficial epithelium was excluded from this analysis. Based on the original Geboes scoring system, Grade 3 was classified as follows:

No Infiltration: Grade 3.0;

<5% of crypts involved: Grade 3.1;

<50% of crypts involved: Grade 3.2;

>50% of crypts involved: Grade 3.3.

### 2.5. Raters

Two raters performed the Komagane evaluation method of the Geboes score Grade 3. The raters had different durations of experience in performing and interpreting histological evaluations (28 years for the expert rater, and 6 months for the beginner rater). Both raters had extensive training (including written definitions, visual depictions, and verbal explanations) regarding the reliable and consistent use of the Komagane evaluation method for the Geboes score Grade 3.

### 2.6. Reliability Testing

The intra-rater test–retest reliability, the inter-rater reliability, and the construct validity were evaluated for the Komagane evaluation method for the Geboes score Grade 3, which was obtained for all glass slides by the same two raters, 4 weeks apart, and with the order of slide presentations randomized.

### 2.7. Statistical Analysis

Data are presented as the means and standard deviations or the median [interquartile range (IQR)]. Statistical tests were employed to compare the results of two groups. The χ^2^-test (with Yates’ correction for continuity, where appropriate) was used for the comparisons of categorical data. Fisher’s exact test was used when the numbers were small. For parametric data, Student’s *t*-test was used when two means were compared. For nonparametric data, the Mann–Whitney rank sum test was used when two medians were compared. A two-sided *p*-value < 0.05 was considered statistically significant. Statistical analyses were conducted using EZR (Jichi Medical University, Saitama, Japan).

Kappa statistics were used to assess intra- and inter-rater reliability and construct validity. To assess intra-rater reliability, 30 paired ratings were carried out; then, a weighted Kappa [10] was calculated to account for the level of disagreement, with comparisons made on the same slide four weeks apart. A similar approach was used to examine the construct validity by comparing the initial scores with those of the criterion standard provided by the same two raters. The inter-rater reliability was determined using a multi-rater Kappa statistic [10], which measures the degree of agreement between raters for each of the four categories (Grades 3.0, 3.1, 3.2, and 3.3). A weighted average of the category-specific agreements was then calculated, with weights based on the number of ratings in each category, yielding the Kappa value.

## 3. Results

### 3.1. Patients’ Clinical Characteristics

The clinical features of the 30 patients with active UC are summarized in Table 1. There were 20 men and 10 women, with a median age of 46 years (IQR: 37–57 years) and a median illness duration of 0 years (IQR: 0–2 years). The types of disease extension were total colitis in 18 patients, left-sided colitis in 7 patients, and proctitis in 5 patients. Among the 30 patients, 11 (37%) had a pre-existing diagnosis of UC, while 19 (63%) were newly diagnosed with UC based on the findings of this examination.

At study enrollment, the median scores of the clinical activities were as follows: CAI—eight points; numeric rating scale (for bowel urgency)—six points. The distribution of the MES was as follows: MES 1—11 patients (37%); MES 2—18 patients (60%); and MES 3—1 patient (3%). Among the eleven patients with a pre-existing diagnosis of UC, five received 5-aminosalicylic acid (5-ASA) monotherapy, one received sulfasalazine monotherapy, one received a combination therapy of prednisolone and 5-ASA, and one received a combination of Janus kinase inhibitor and 5-ASA. The remaining three patients received no medication related to UC.

### 3.2. Reliability Testing for the Komagane Evaluation Method

The intra-rater test–retest reliability for the Komagane evaluation method, assessed four weeks apart, was determined using a weighted Kappa statistic. The analysis showed perfect agreement for both the expert rater (κ = 1.0) and the beginner rater (κ = 1.0), indicating excellent consistency of the scoring method for each individual evaluator. The construct validity was also 100% for both raters. The inter-rater reliability between the two raters for the Komagane evaluation method was substantial, with a multi-rater Kappa value of 0.85.

### 3.3. The Distribution of the Geboes Score Grade 3

An evaluation of the Geboes score Grade 3 revealed the following distribution: six patients (20%) were classified as Grade 3.0, seven (23%) were classified as Grade 3.1, sixteen (53%) were classified as Grade 3.2, and one (3%) was classified as Grade 3.3 (Table 2).

**Figure 2:**
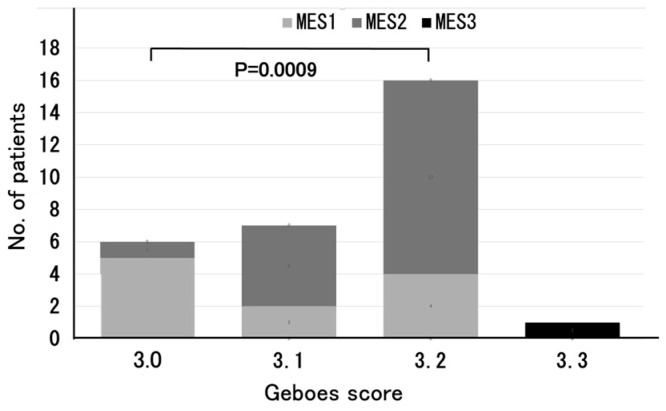
Distribution of MES 1, 2, or 3 in relation to Geboes score Grades 3.0, 3.1, 3.2, and 3.3. Abbreviations—MES: Mayo endoscopic subscore.

**Figure 3:**
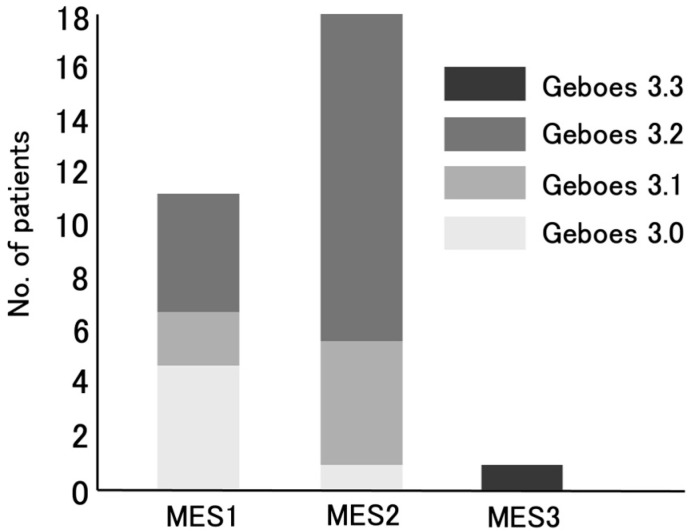
Distribution of Geboes score Grades 3.0, 3.1, 3.2, and 3.3 in relation to MES 1, 2, or 3. Abbreviations—MES: Mayo endoscopic subscore.

In addition, among the six patients classified into the Grade 3.0 group, the majority (83%) had an MES of one. Five of the seven patients classified as Grade 3.1 reported an MES of two (71%), while twelve out of the sixteen patients classified as Grade 3.2 (75%) reported an MES of two. A significant difference in MES was observed between the Grade 3.0 and Grade 3.2 groups (*p* = 0.0009) (Figure 2). Of the 18 UC patients with MES 2, 5 (28%) were classified as Grade 3.1, and 12 (67%) were classified as Grade 3.2 (Figure 3).

**Figure 4:**
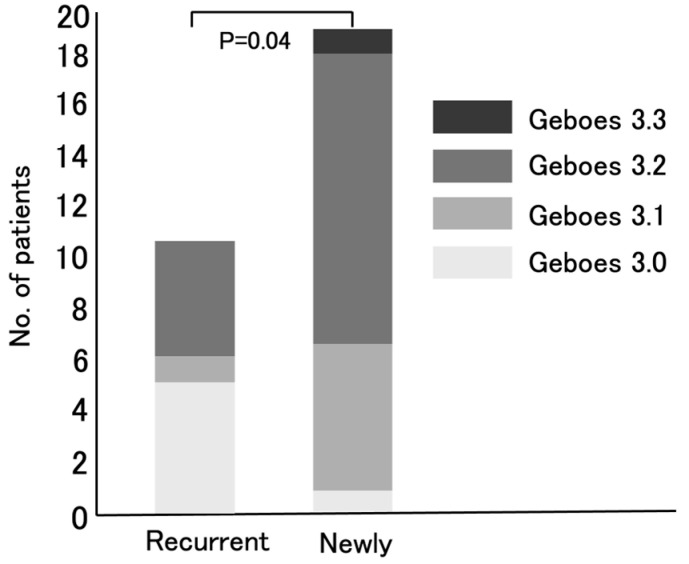
Comparison of the distribution of Geboes score Grades 3.0, 3.1, 3.2, and 3.3 between recurrent and newly diagnosed ulcerative colitis patients.

The distribution of the Geboes score Grade 3 was then compared between patients with recurrent UC and those newly diagnosed with UC (Figure 4). Baseline characteristics (age, sex, disease duration, and CAI) were similar between recurrent UC and newly diagnosed UC patients. In particular, the MES scores were similar between the two groups (*p* = 0.099). Therefore, the significant difference in the Geboes Grade 3 scores is unlikely to be due to these factors. Among the eleven recurrent UC patients, five (45%), one (9%), and five (45%) were classified into the Grade 3.0, Grade 3.1, and Grade 3.2 groups, respectively. In contrast, among the nineteen newly diagnosed patients, one (5%), six (32%), eleven (58%), and one (5%) were classified as having Grade 3.0, Grade 3.1, Grade 3.2, and Grade 3.3 epithelial neutrophilic infiltration, respectively. There was a significant difference in neutrophilic infiltration between the two groups (*p* = 0.04).

### 3.4. Changes in Clinical and Endoscopic Activity from Before to After Mirikizumab Treatment

**Figure 5:**
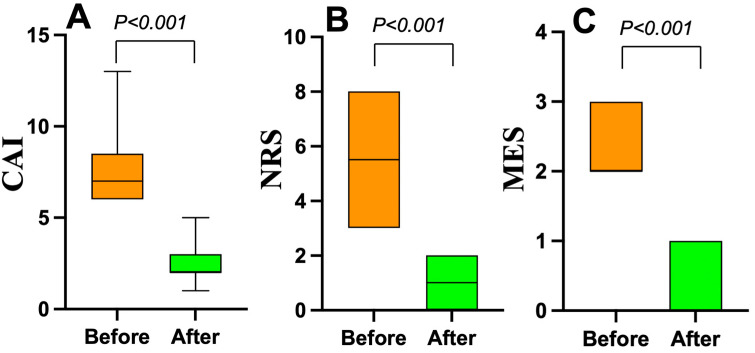
The changes in ten active ulcerative colitis patients’ clinical activity before and after mirikizumab induction therapy. (**A**): Clinical activity index (CAI) scores. (**B**): Numeric rating scale (NRS) values for bowel urgency. (**C**): Mayo endoscopic subscore (MES) data.

Among the thirty patients, ten who were endoscopically active and who exhibited pathologically high neutrophil infiltration in the epithelium of the colonic mucosa (Geboes score ≥ 3.2) were administered mirikizumab. Nine of the ten patients received 300 mg of mirikizumab administered intravenously every 4 weeks for 12 weeks. The remaining patient was treated with 300 mg of mirikizumab intravenously every 4 weeks for 24 weeks. The changes in the patients’ clinical and endoscopic activity from before to after mirikizumab induction therapy are illustrated in Figure 5. The median CAI values before and after mirikizumab treatment were seven and two, revealing a significant decrease in the CAI (*p* < 0.001). The NRS scores also decreased significantly from 5.5 to 1 (*p* < 0.001). The median MES changed from two to zero points after mirikizumab treatment (*p* < 0.001).

## 4. Discussion

In this study, we presented a simple and reproducible method for the evaluation of the Geboes score Grade 3. A high neutrophilic infiltration within crypts, corresponding to Grade 3.2 or 3.3, was observed in approximately 60% of the enrolled UC patients. Additionally, endoscopic mucosal activity was correlated with the histological findings, suggesting that more severe mucosal inflammation was associated with increased neutrophilic infiltration in the epithelium. Our study improves the diagnostic accuracy for UC patients by developing a reproducible subclassification of the Geboes score Grade 3, with potential future implications for prognostication—particularly in identifying IL-23 antagonist responders—although the current focus remains on refining the consistency of the histopathological assessment. To our knowledge, there are currently no published studies demonstrating a direct correlation between the Geboes score Grade 3 and IL-23 levels. Our study is exploratory in this regard, aiming to provide a histological framework that may facilitate such future investigations.

The relationship between the Geboes score Grade 3 and MES is particularly noteworthy. Most of the patients with Grade 3.0 reported an MES of 1, whereas those with Grades 3.1 and 3.2 more frequently reported an MES of 2. As shown in Figure 2, there was a significant difference in MES between the Grade 3.0 and Grade 3.2 groups (*p* = 0.0009), reinforcing previous reports in which the Geboes score and MES were positively correlated, with greater mucosal inflammation reflecting more severe histological inflammation [11]. These findings support the idea that MES, which is commonly used for evaluating endoscopic activity, may complement the histological assessment for a more comprehensive evaluation of UC disease activity.

From an immunological perspective, the role of Th17 in chronic inflammation is well-established. Th17-driven cytokine production mobilizes and activates neutrophils, contributing to sustained inflammation [4,5]. In active UC, inflammatory cytokine profiles evolve over time, with Th1-associated cytokines being predominant in the early phase and Th2-related cytokines being predominant in the late phase. On the other hand, Th17-associated cytokines are elevated in both the early and late phases, suggesting their continuous involvement [4]. Based on the evaluation of the Geboes score Grade 3, the activation of Th17 in UC patients can be predicted. Notably, newly diagnosed UC patients exhibited significantly higher Grade 3 scores than recurrent UC patients (*p* = 0.04) (Figure 4). This may mean that the number of active UC patients in whom IL-23 exerts its effects by activating Th17 cells, resulting in high neutrophil infiltration in the colonic mucosal epithelium, has been increasing in Japan. In other words, the number of active UC patients likely to respond effectively to IL-23 antagonists may be growing.

The MES scores were similar between newly diagnosed and relapsed UC patients (*p* = 0.099). Therefore, the significant difference in Geboes Grade 3 scores is unlikely to be related to the MES. As 73% of patients were untreated prior to the study and only 2/30 were on immunosuppressants (one was taking prednisolone and one was taking Janus kinase inhibitor), systemic drug effects are unlikely. These findings suggest that the differences in inflammation between the two groups are due to intrinsic disease biology.

To ensure a straightforward and reproducible assessment of the Geboes score Grade 3, a simplified methodology was developed in this study. Although relatively high inter-observer variability has been reported for Grades 3 and 4 [7], the original description of the Geboes score lacked detailed criteria for counting crypts and neutrophilic infiltration [6]. As the inter-rater reliability for this Komagane evaluation method was 0.85 (Kappa value), we believe that we have developed an accessible and reproducible method. Therefore, we expect that even non-expert pathologists could obtain reliable results using the Komagane evaluation method for the Geboes score Grade 3, thereby improving their identification of UC patients who are likely to respond effectively to IL-23 antagonists in routine clinical practice. Actually, one of the IL-23 antagonists, mirikizumab treatment, provided clinical and endoscopic improvements in active UC 10 patients whose Geboes score was ≥3.2 in the Komagane evaluation method of the Geboes score Grade 3.

In our study, the term “Th17 activation” specifically refers to the IL-23-driven pathogenic Th17 activity that is associated with epithelial neutrophilic infiltration (Grades 3.2/3.3) [12]. While non-pathogenic Th17 cells may coexist in the mucosa, their IL-10-mediated immunosuppressive functions are unlikely to drive the neutrophilic infiltration that is central to our scoring system [13]. While our focus on Grade 3 identifies epithelial-phase Th17 activity, Grade 2B lamina propria neutrophils may represent earlier IL-23/Th17 signaling that precedes epithelial invasion. Our study focused only on Grade 3 in order to clarify the reproducibility of the association between neutrophil infiltration and IL-23 levels in UC patients.

Although immunohistochemistry is a valuable technique for the direct visualization of IL-23, the primary objective of this study was to refine the existing Geboes score Grade 3 to enhance consistency in routine clinical practice, particularly for pathologists who may not have immediate access to immunohistochemistry or for whom it is not a standard procedure. Therefore, the expression of IL-23 was not provided by immunohistochemistry.

This study has several limitations. It was conducted at a single hospital in Japan with a small number of patients (*n* = 30). The small sample size and the use of the MES as the main comparator limit the strength of our conclusions. Future research should compare the Komagane method with other histological scoring systems and biomarkers. Although we tried to reduce the selection bias by including all consecutive patients with active UC over one year, the results may not be widely applicable. Therefore, this study should be considered as being preliminary. Future studies should use this scoring method in treatment trials to see whether it can predict drug responses. Larger prospective studies with longer follow-up times are needed to confirm our findings and to evaluate the benefits of combining histological and endoscopic assessments in UC.

## 5. Conclusions

We have developed a simple and reproducible method for the evaluation of the Geboes score Grade 3. When applying this method to a group of 30 patients, we found that approximately 60% were classified as having Grade 3.2 epithelial neutrophilic inflammation or higher based on the reproducible scoring method. This method may help to identify UC patients who are likely to respond effectively to IL-23 antagonists.

## Figures and Tables

**Table 1:** Clinical characteristics of the enrolled subjects (*n* = 30).

Males/females, *n* (%)	20 (67)/10 (33)
Age, yrs; median (IQR)	46 (37–57)
Disease duration, yrs; median (IQR)	0 (0–2)
Disease extension, *n* (%)	
Proctitis	5 (17)
Left-sided colitis	7 (23)
Total colitis	18 (60)
Disease course, *n* (%)	
First episode	19 (63)
Recurrent	11(37)
C-reactive protein, mg/dL; median (IQR)	0.04 (0.02–0.81)
Serum LRG, mg/mL; median (IQR)	17 (16–19)
CAI, median (IQR)	8 (6–13)
NRS, median (IQR)	6 (4–9)
MES0, *n* (%)	0 (0)
MES1, *n* (%)	11 (37)
MES2, *n* (%)	18 (60)
MES3, *n* (%)	1 (3)
Medication before entry, *n* (%):	
None	22 (73)
5-ASA	5 (17)
Sulfasalazine	1 (3)
Prednisolone and 5-ASA	1 (3)
Janus kinase inhibitor and 5-ASA	1 (3)

IQR: interquartile range; SD: standard deviation; LRG: leucine-rich α2-glycoprotein; CAI: clinical activity index; NRS: numeric rating scale (for bowel urgency); MES: Mayo endoscopic subscore; 5-ASA: 5-aminosalicylic acid.

**Table 2:** Geboes score of the enrolled subjects (*n* = 30).

Grade 3.0	No infiltration	6 (20%)
Grade 3.1	<5% of crypts involved	7 (23%)
Grade 3.2	<50% of crypts involved	16 (53%)
Grade 3.3	>50% of crypts involved	1 (3%)

The percentage of crypts with neutrophil infiltration was calculated based on the Komagane evaluation method for Geboes score Grade 3.

## Data Availability

The data underlying this article will be made available upon reasonable request to the corresponding author.
